# Nonlinear association between age and verbal fluency and moderation by adiposity: cross-sectional evidence from the Look Up 8+ study

**DOI:** 10.3389/fnagi.2026.1912213

**Published:** 2026-07-29

**Authors:** Giulia Giordano, Luca Mastrantoni, Riccardo Calvani, Roberta Terranova, Federica D’Ignazio, Antonella Risoli, Francesco Landi, The Look Up 8+ Study Group, Francesco Landi, Roberto Bernabei, Emanuele Marzetti, Riccardo Calvani, Luca Mariotti, Stefano Cacciatore, Hélio José Coelho-Junior, Francesca Ciciarello, Vincenzo Galluzzo, Anna Maria Martone, Anna Picca, Andrea Russo, Sara Salini, Matteo Tosato, Gabriele Abbatecola, Clara Agostino, Fiorella Ambrosio, Francesca Banella, Carolina Benvenuto, Damiano Biscotti, Vincenzo Brandi, Maria Modestina Bulla, Caterina Casciani, Lucio Catalano, Camilla Cocchi, Giuseppe Colloca, Federica Cucinotta, Emanuela D’Angelo, Mariaelena D’Elia, Federica D’Ignazio, Daniele Elmi, Marta Finelli, Francesco Pio Fontanella, Domenico Fusco, Ilaria Gattari, Giordana Gava, Tommaso Giani, Giulia Giordano, Rossella Giordano, Francesca Giovanale, Simone Goracci, Silvia Ialungo, Rosangela Labriola, Elena Levati, Myriam Macaluso, Luca Marrella, Claudia Massaro, Rossella Montenero, Maria Vittoria Notari, Maria Paudice, Martina Persia, Flavia Pirone, Simona Pompei, Rosa Ragozzino, Carla Recupero, Antonella Risoli, Stefano Rizzo, Daria Romaniello, Giulia Rubini, Barbara Russo, Stefania Satriano, Giulia Savera, Elisabetta Serafini, Annalise Serra Melechì, Francesca Simeoni, Sofia Simoni, Chiara Taccone, Elena Tagliacozzi, Roberta Terranova, Salvatore Tupputi, Matteo Vaccarella, Emiliano Venditti, Chiara Zanchi, Maria Zuppardo

**Affiliations:** 1 Department of Geriatrics, Orthopedics and Rheumatological Sciences, Fondazione Policlinico Universitario Agostino Gemelli IRCCS, Rome, Italy; 2 Medical Oncology Unit, Fondazione Policlinico Universitario Agostino Gemelli IRCCS, Rome, Italy; 3 Università Cattolica del Sacro Cuore, Rome, Italy

**Keywords:** adiposity, ageing, chair stand test, moderation analysis, physical activity, verbal fluency, waist-height ratio

## Abstract

**Background:**

Verbal fluency (VF) declines with age, but the shape of this association and its modifiers remain debated. In community-dwelling adults aged 30–80 years from the Look Up 8+ study, we investigated the nonlinear association between age and phonemic VF and evaluated the impact of educational attainment, lifestyle factors, central adiposity, and muscle function on VF performance.

**Methods:**

Verbal fluency was assessed using a 30-s phonemic fluency task. Generalized additive models (GAMs) with negative binomial distribution characterized nonlinear age-related trajectories. Sequential models adjusted for sex, educational attainment, physical activity (PA), healthy diet, smoking status, blood pressure, total cholesterol, blood glucose, body mass index, and sleep quality. Central adiposity was evaluated using waist-height ratio (WHtR), with waist-hip ratio (WHR) included in sensitivity analyses. Moderation analyses tested whether central adiposity modified the association between age and VF. Associations between VF and muscle- parameters were also examined.

**Results:**

Among 3,615 adults (54% female; median age 58 years; median VF score 10 words), the age-VF association was significantly nonlinear (edf = 3.36, *p* < 0.001), peaking at 45–50 years and showing progressively lower values thereafter. Higher educational attainment was strongly associated with greater VF: high school/BSc (IRR = 1.218, 95% CI 1.168–1.271, *p* < 0.001) and master’s/postgraduate (IRR = 1.326, 95% CI 1.269–1.385, *p* < 0.001) versus lower education. Healthy diet (IRR = 1.044, *p* < 0.001), PA (IRR = 1.038, *p* = 0.005), and normal blood glucose (IRR = 1.050, *p* = 0.022) were independently associated with higher VF; the dietary association was confirmed for Mediterranean diet adherence. Higher central adiposity was associated with lower VF for both WHtR (IRR = 0.973, *p* < 0.001) and WHR (IRR = 0.979, *p* = 0.003) and significantly moderated the age-VF association (Age × WHtR: IRR = 0.985, *p* = 0.014; Age × WHR: IRR = 0.986, *p* = 0.021). Among muscle-related parameters, longer chair stand test time was associated with lower VF (IRR = 0.964, *p* < 0.001).

**Conclusion:**

Age and VF are nonlinearly associated across adulthood, with educational attainment being a major determinant. Higher central adiposity is associated with lower VF and moderates the associated with age. Lower-limb functional performance was independently associated with VF, potentially reflecting a functional pathway linking adiposity, mobility, and cognitive aging.

## Introduction

Cognitive function decline represents one among several aspects in aging process (Harada et al., 2013). As the world population is growing older, brain aging and cognitive impairment are currently among the most investigated aspects: a preserved cognitive function represents a key component in successful healthy aging, as brain health contributes significantly on activities of daily living (ADL) preservation and subsequently on the ability to live independently (Imboden, 2024). Growing evidence is focusing on cognitive function decline and potential protective and risk factors: omics data analysis and studies on molecular pathway hold great potential in developing potential healthy brain aging strategies (Myung-Hoon et al., 2026; Navakkode and Kennedy, 2024; Oh et al., 2025).

To assess cognitive function, several tools and scale can be used both in clinical practice and in clinical research context: verbal fluency (VF), defined as the ability to rapidly produce words within specific conditions, and has been confirmed to change during lifespan, represent a useful resource, providing insights of a subject’s cognitive abilities, including working memory (Aita et al., 2019; Villalobos et al., 2023). VF is one among cognitive abilities most sensitive to aging, and its decline represents an early marker of neurodegenerative diseases onset (McDonnell et al., 2020; Shao et al., 2014). Its performance can be evaluated through the phonemic and the semantic fluency (Harrison et al., 2000). Phonemic fluency represents capacity to generate words beginning with a specific letter, while semantic fluency refers to being able to name different items within a specific category. Despite its high sensitivity and ease of use, several aspects regarding VF trajectories during the lifespan and in the aging process require further evidence, including the shape of VF trajectory, and potential impact of cognitive reserve, social interactions, education, and lifestyle behaviors.

In addition, the relationship between cognitive performance and body composition is progressively gaining attention: central adiposity, assessed by waist-height ratio (WHtR) and waist-hip ratio (WHR) is emerging as a stronger predictor of cardiovascular and cognitive outcomes than body mass index (BMI), given its direct reflection of visceral fat accumulation (Ashwell et al., 2012; Chen et al., 2023; Filgueiras et al., 2019; Huxley et al., 2010; Paniagua et al., 2008). In addition, aging itself is associated with visceral adiposity and organs’ fat storage increase in several organs, including muscle (Ponti et al., 2020). Adipose tissue drives the adipose-related chronic low-grade inflammation, along with concurrent biological pathways including insulin resistance, and vascular dysfunction, all linking abdominal adiposity to cognitive decline (Nguyen et al., 2014). Beyond adiposity and muscle mass, lifestyle factors such as physical activity (PA) and healthy dietary patterns have been proposed as independent, non-pharmacological modulators of cognitive aging. Regular PA is thought to support cognitive function partly through the muscle–brain axis, whereby skeletal muscle acts as an endocrine organ releasing myokines with autocrine, paracrine, and endocrine effects on the central nervous system (Hoffmann and Weigert, 2017; Vints et al., 2023). Similarly, adherence to dietary patterns enriched in mono-unsaturated fatty acids and polyphenols has been associated with better memory and verbal fluency performance in older adults (Lopez de Coca et al., 2025). Concurrently, skeletal muscle decline represents a parallel aging pathway, with established links to cognitive function (Clark and Manini, 2012; Cruz-Jentoft et al., 2010; Peng et al., 2020). Whether these conditions operate independently or synergistically to modulate age-related cognitive decline is a matter of interest.

In this study, we aimed to evaluate (1) the nonlinear association between age and VF in community dwelling adults, (2) the impact of educational attainment, PA and diet on VF, (3) the association between central adiposity and VF and its moderating effect of the age-VF association, and (4) the association between muscle function and VF.

## Materials and methods

### Study design and participants

The Look Up 8+ study (Longevity Check Up) is an ongoing initiative, started on June 1st 2015, and supported by Università Cattolica del Sacro Cuore- Department of Geriatrics and Fondazione Policlinico Universitario “Agostino Gemelli” IRCCS (Rome, Italy). The study protocol received approval from the Ethical Committee of Università Cattolica del Sacro Cuore (Protocol No. A.1220/ce/2011). Further details are provided in previously published papers (Giordano et al., 2025; Landi et al., 2018).

The Look Up 8+ study is conducted in non-conventional settings in Italy and started on June 1st, 2015. Study participants were recruited in non-clinical settings including shopping centers, social gatherings and prevention campaigns organized by Università Cattolica del Sacro Cuore. All participants were ≥18 years old and provided written informed consent prior to registration. Exclusion criteria were self−reported pregnancy, inability to perform the required physical performance tests, refusal to undergo capillary blood test and inability or unwillingness to provide written informed consent.

The present manuscript was prepared in accordance with the Strengthening the Reporting of Observational Studies in Epidemiology (STROBE) reporting guidelines for observational studies (von Elm et al., 2007). The STROBE checklist can be found in Supplementary material.

### Measurements of variables and endpoints

Verbal fluency was the outcome of interest and age was considered as primary exposure, with education, central adiposity (assessed via WHtR/WHR), lifestyle/cardiometabolic factors, and muscle function evaluated as independent covariates influencing VF. Physical activity, diet and central adiposity were also evaluated as moderators of the age-VF association.

Verbal fluency was assessed using the using the phonemic fluency paradigm, in which participants had to produce as many words beginning with “F” letter as possible within 30 s (Shao et al., 2014). The total count of correct, non-repeated responses constituted the outcome variable. City names and personal names were considered as incorrect words. Participants’ answers were accordingly rated for correctness, assuring noticed or unnoticed repetitions, wrong categories, and including morphological variants as correct. Given the operational constraints of the Look Up 8+ community screening setting, the standard 60-s phonemic fluency paradigm was adapted to a 30-s administration window, which have been shown to reliably predict full 60-s totals and to preserve equivalent discriminative accuracy for cognitive impairment (Kaufman et al., 2024). Accordingly, throughout this manuscript the term verbal fluency refers to this brief single-letter phonemic fluency measure.

Years of education were categorized into three levels: low (≤8 years, corresponding to primary and lower-secondary completion), medium (9–16 years, upper-secondary and undergraduate/bachelor’s degree), and high (≥17 years, graduate/master’s degree or postgrad). Regular PA was defined as engaging in exercise or a minimum of 30 min at least twice per week over the preceding year. Cardiovascular health metrics (HM) included healthy diet, smoking (intended as non-active smoker), normal BMI, normal blood pressure profile, normal total cholesterol values, normal blood glucose values and a good sleep quality. Each component was scored 1 (if in range) or 0 (if out of range). A sensitivity analysis was conducted considering Mediterranean Diet (MD) adherence, which was evaluated based on MEDI-LITE score and categorized as low, medium and high adherence.

Waist-to-height ratio, derived from ratio of waist circumference and height, was selected as the primary index of central adiposity (Ashwell et al., 2014). A sensitivity analysis was also conducted considering waist-hip ratio (WHR) in the fully adjusted model. Due to multicollinearity, BMI was not included in these analyses.

Five muscle function parameters were included: appendicular skeletal muscle mass (ASM, kg), handgrip strength (kg), chair stand test (CST) performance (time to perform five consecutive sit-to-stand repetitions, seconds, with higher values indicating poorer performance), calf circumference (CC, measured in centimeters), and “Probable sarcopenia,” based on the European Working Group on Sarcopenia in Older People 2019 criteria (EWGSOP2) (Cruz-Jentoft et al., 2019).

### Statistical analysis

The characteristics of the study subjects were summarized using descriptive statistics. Continuous variables were expressed as median values with interquartile ranges (IQR), and categorical variables were reported as absolute counts and percentages. Descriptive statistics were stratified by sex. Group differences were assessed with Mann-Whitney U tests for continuous variables and chi-squared tests for categorical variables. Sequential regression models were structured to evaluate the primary effect of age, education, central adiposity, lifestyle/cardiometabolic factors, and muscle function.

Given VF as a count outcome, we compared three distributional families for the regression model: Gaussian, Poisson, and negative binomial (NB). To investigate the non-linear associations between age and verbal function, we used generalized additive models (GAMs), fitted using penalized thin-plate regression splines with restricted maximum likelihood (REML) estimation, using mgcv package. Age was modeled as a smooth term using thin plate regression splines (TPRS). Three sequential models were considered: (1) Age smooth alone; (2) Age smooth + education + sex; and (3) model 2 + covariate correction for PA, healthy diet, being non-smoker, normal blood pressure, normal total cholesterol, normal blood glucose, normal BMI and sleep quality. Model fit was examined through quantile-residual diagnostics (QQ-plot, residuals versus linear predictor, residual distribution, and observed-versus-fitted values). Concurvity was assessed to evaluate non-linear dependencies among model terms. Multicollinearity was assessed using generalized variance inflation factors. Non-linearity was evaluated considering the effective degrees of freedom (edf) and basis-dimension adequacy was evaluated by comparing the edf with the basis dimension (k’), with sensitivity analyses refitting the age smooth at increasing k. Overdispersion was assessed by comparing the Pearson dispersion statistic under Poisson and negative binomial families. To quantify the rate of verbal fluency decline, the first derivative - representing the marginal rate of change in the outcome per unit change in age - was computed using the gratia package. Significant zero crossings of derivatives analysis were used to identify periods of significant change.

To test whether WHtR (or WHR), and PA moderated the age-related decline in VF, we fitted a NB model including Age × WHtR (Age × WHR), and Age × PA interaction, adjusting for the same covariates except for BMI (to avoid collinearity) and sleep quality. A Johnson-Neyman (JN) analysis was performed using the interactions R package to identify the range of WHtR values at which the simple slope of age on VF was statistically significant (Bauer and Curran, 2005). To assess whether the association between central adiposity and VF varied nonlinearly with age, we fitted varying-coefficient negative binomial GAMs in which a smooth-by-age term allowed the effect of central adiposity (WHtR or WHR) to vary smoothly across age, adjusting for the same covariates as the linear interaction model. Continuous moderators were centered and scaled for moderation analysis. Regression coefficients with 95% confidence intervals were reported. Models were compared using AIC, BIC, RMSE, and R^2^ Analyses were performed in R (vers.4.6.0). Statistical significance was defined at a two-sided *p*-value < 0.05.

## Results

### Sample characteristics

A total of 3,615 adults (1,963 female, 54%) were included. Median age was 58 years (IQR: 49–66). Median VF score was 10 words (IQR: 7–12). Education years was distributed as follows: primary-secondary school (≤8 years): 13%; high school or bachelor’s degree (9–16 years): 44%; master’s degree or postgrad (≥17 years): 44%. Most participants were physically active (*n* = 2,322, 64%), non-smokers (*n* = 3,023, 84%), adhered to a healthy nutritional regimen (*n* = 2,198, 61%), reported normal blood glucose values (*n* = 3,265, 90%), normal BMI (*n* = 2,188, 61%) and a good sleep quality (*n* = 2,491, 69%). A total of 1,919 subjects (53%) reported altered cholesterol levels. Median handgrip strength was 29 kg (24–38), 25 (21–28) in females and 39 (34–45) in males, while median chair stand test performance was 7.2 s (6.0–8.6). Probable sarcopenia (EWGSOP2 criteria) was present in 231 participants (6.4%). Median waist-to-height ratio was 0.52 (IQR: 0.47–0.57); males (0.53, IQR: 0.49–0.58) showed higher central adiposity than females (0.50, IQR: 0.45–0.57, *p* < 0.001). Full baseline characteristics are presented in Table 1.

**TABLE 1:** Patients’ characteristics in the overall cohort.

Characteristic	Overall *N* = 3615	Female *N* = 1963	Male *N* = 1652	*P*-value
Age (years)	58 (49–66)	57 (49–66)	58 (49–67)	0.53
Verbal fluency (score)	10 (7–12)	10 (8–12)	10 (7–12)	0.019
Education (years)				0.33
Primary-secondary school (≤8 yr)	453 (13%)	260 (13%)	193 (12%)	
High school - bachelor’s (9–16 yr)	1587 (44%)	848 (43%)	739 (45%)	
Master’s degree - postgrad (≥17 yr)	1575 (44%)	855 (44%)	720 (44%)	
Waist-to-height ratio	0.52 (0.47–0.57)	0.50 (0.45–0.57)	0.53 (0.49–0.58)	<0.001
Physical activity				<0.001
Inactive	1293 (36%)	872 (44%)	421 (25%)	
Active	2322 (64%)	1091 (56%)	1231 (75%)	
MEDI-LITE adherence				0.27
Low (≤8)	826 (24%)	437 (23%)	389 (24%)	
Moderate-high (>8)	2673 (76%)	1473 (77%)	1200 (76%)	
Healthy diet				<0.001
No healthy diet	1417 (39%)	624 (32%)	793 (48%)	
Healthy diet	2198 (61%)	1339 (68%)	859 (52%)	
Smoke				0.11
Smoker	592 (16%)	339 (17%)	253 (15%)	
Non-smoker	3023 (84%)	1624 (83%)	1399 (85%)	
Blood pressure				< 0.001
Hypertension	1938 (54%)	873 (44%)	1065 (64%)	
Normal BP	1677 (46%)	1090 (56%)	587 (36%)	
Cholesterol				<0.001
Hypercholesterolemia	1919 (53%)	1205 (61%)	714 (43%)	
Normal	1696 (47%)	758 (39%)	938 (57%)	
Appendicular skeletal muscle mass (kg)	18.5 (14.9–23.6)	15.1 (13.6–16.8)	23.9 (22.3–25.6)	<0.001
Handgrip strength (kg)	29 (24–38)	25 (21–28)	39 (34–45)	<0.001
Chair stand test (s)	7.20 (6.03–8.60)	7.35 (6.10–8.80)	7.07 (6.00–8.39)	<0.001
Sarcopenia (EWGSOP2)				0.001
No	3384 (94%)	1861 (95%)	1523 (92%)	
Yes	231 (6.4%)	102 (5.2%)	129 (7.8%)	
Calf circumference (cm)	36.0 (34.0–38.0)	35.0 (34.0–38.0)	37.0 (35.0–39.0)	<0.001
Blood glucose				0.002
Altered	350 (9.7%)	162 (8.3%)	188 (11%)	
Normal	3265 (90%)	1801 (92%)	1464 (89%)	
BMI (HM)				<0.001
Altered	1427 (39%)	595 (30%)	832 (50%)	
Normal	2188 (61%)	1368 (70%)	820 (50%)	
Sleep quality (HM)				<0.001
Bad	1124 (31%)	681 (35%)	443 (27%)	
Good	2491 (69%)	1282 (65%)	1209 (73%)	

Continuous values are represented as median [IQR]. Categorical variables as *n* (%). Wilcoxon rank sum test was used for continuous variables and Pearson’s Chi-squared test for categorical variables. IQR, interquartile range; N, number; BP, blood pressure; EWGSOP2, European working group on Sarcopenia in Older People 2; HM, health metrics.

### Association between age and verbal fluency

**FIGURE 1:**
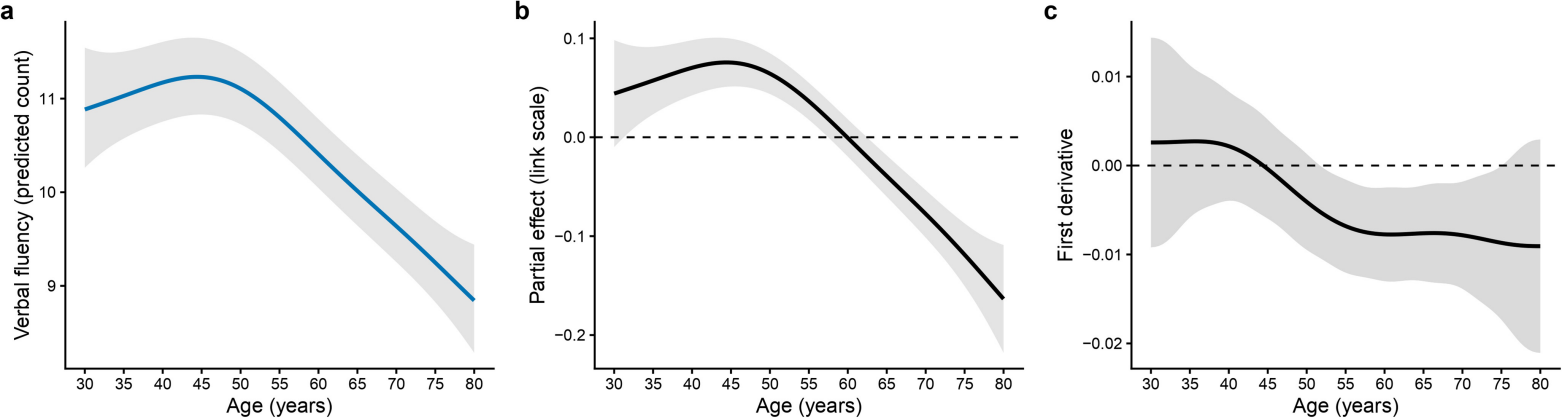
Non-linear association between age and verbal fluency. **(a,b)** Predicted VF scores according to age, original scale **(a)** and partial effect **(b)**. Results from the generalized additive model adjusted for sex, and physical activity, smoking status, healthy diet, education level, blood pressure, cholesterol values, blood glucose, BMI and sleep quality (health metrics) (model 3). Shaded areas represent 95% confidence intervals. **(c)** Rate of change (first derivative) of the association between age and verbal fluency. Values below the dashed zero line indicate age periods associated with a decline in verbal fluency scores. The shaded area represents the 95% confidence interval.

A Negative Binomial family with log link was selected to model verbal fluency, compared to Gaussian and Poisson (Supplementary Table 1). The GAM provided better fit than the linear model, and the addition of covariates provided progressive improvement in model fit (Supplementary Table 2): the fully adjusted GAMl (model 3) had a significant better fit (ANOVA *p* < 0.001) than the fully adjusted linear model and was considered as the primary model (Supplementary Figure 1 and Supplementary Table 3). The age smooth in the primary GAM (model 3) was statistically significant (edf = 3.36, *p* < 0.001), confirming substantial non-linearity in the association between age and verbal fluency. Predicted verbal fluency reached the maximum at approximately 45–50 years, with progressively lower values thereafter (Figures 1a, b). The first derivative of age smooth showed three distinct moments: between ages 30 and 45 years the first derivative was positive, indicating a period of relative stability/increase, between 45 and 60 we observed a sharp change in the derivate, from positive to negative, and then after 60 years it became significantly negative reaching a plateau, corresponding to a stable decline (Figure 1c). No significant main effect of sex on VF was observed.

### Education, lifestyle and cardiovascular factors

**FIGURE 2:**
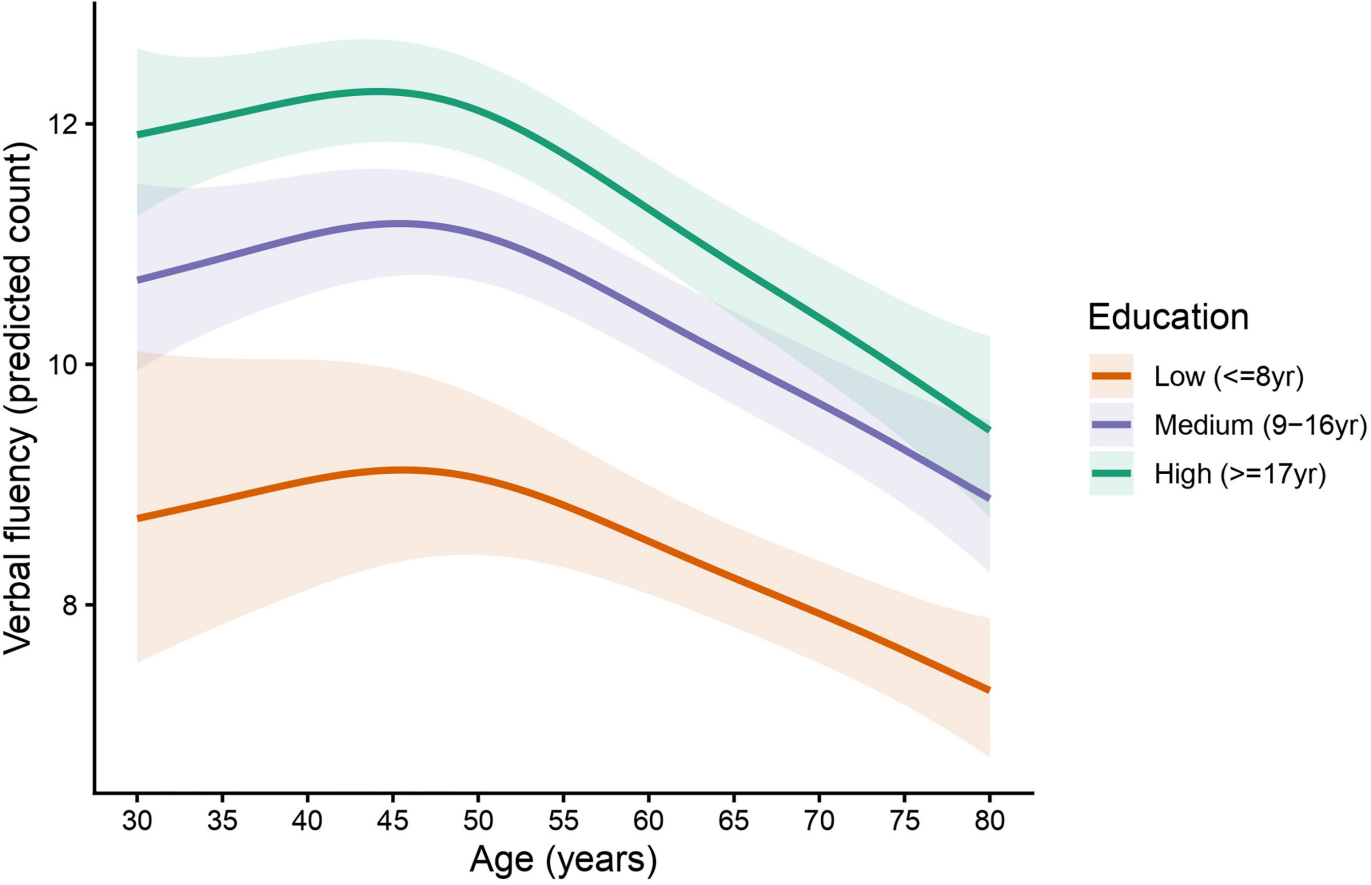
Non-linear association between age and verbal fluency scores, stratified by educational attainment. Results are adjusted for sex, physical activity, smoking status, healthy diet, education level, blood pressure, cholesterol values, blood glucose, BMI and sleep quality (health metrics) (model 3). Shaded areas represent 95% confidence intervals.

Educational attainment exerted a strong independent effect on verbal fluency after adjustment for age, sex, and all lifestyle covariates (Table 2). Using primary-secondary school as reference, subjects with high school - BSc education showed 21.8% higher verbal fluency (IRR = 1.218, 95% CI 1.168–1.271, *p* < 0.001), and those with master’s degree (≥17 years) showed 32.6% higher verbal fluency (IRR = 1.326, 95% CI 1.269–1.385, *p* < 0.001). Education-stratified VF values according to age confirmed that the age smooth was characterized by an inverted-U shape, with a period of relative stability or slight increase in early midlife followed by steeper age-related differences after approximately age 50 (Figure 2).

**TABLE 2:** Association of educational attainment and lifestyle/cardiometabolic factors with verbal fluency.

Variables	Healthy diet (HM) (*n* = 3615)		Mediterranean diet (MEDI-LITE) (*n* = 3499)	
	Est.	*P*-value	Est.	*P*-value
Education (9–16 yr)	1.218 (1.168–1.271)	<0.001	1.220 (1.170–1.273)	<0.001
Education (≥17 yr)	1.325 (1.269–1.384)	<0.001	1.332 (1.275–1.392)	<0.001
Sex (male)	0.989 (0.963–1.014)	0.380	0.982 (0.957–1.007)	0.160
Physically active (HM)	1.038 (1.012–1.065)	0.004	1.039 (1.012–1.066)	0.004
Non-smoker (HM)	1.013 (0.982–1.046)	0.420	1.014 (0.982–1.047)	0.390
Healthy diet (HM)	1.044 (1.019–1.070)	<0.001	–	–
Blood pressure normal (HM)	1.020 (0.994–1.046)	0.138	1.023 (0.996–1.050)	0.091
Cholesterol normal (HM)	0.992 (0.967–1.016)	0.497	0.990 (0.965–1.015)	0.408
Blood glucose normal (HM)	1.050 (1.007–1.095)	0.023	1.043 (1.000–1.089)	0.053
BMI normal (HM)	1.023 (0.997–1.049)	0.086	1.028 (1.001–1.055)	0.038
Sleep quality good (HM)	1.002 (0.977–1.028)	0.889	1.007 (0.981–1.033)	0.620
MEDI-LITE adherence moderate-high	–	–	1.053 (1.024–1.083)	<0.001

Incidence rate ratios for educational attainment and lifestyle/cardiometabolic factors on verbal fluency, from the fully adjusted negative binomial model. For health metrics, coefficients are referred to the positive value (in range). Estimates and confidence intervals are exponentiated. Est, Estimate; yr, years; HM, health metrics; AIC, Akaike Information Criterion; BIC, Bayesian Information Criterion.

Among the lifestyle and cardiometabolic covariates included in model, three factors showed statistically significant independent associations with verbal fluency. Healthy diet was associated with a 4.4% higher verbal fluency score (IRR = 1.044, 95% CI 1.018–1.070, *p* < 0.001), PA was associated with a 3.8% higher verbal fluency (IRR = 1.038, 95% CI 1.012–1.066, *p* = 0.005), and normal blood glucose values showed a 5.0% higher verbal fluency (IRR = 1.050, 95% CI 1.007–1.095, *p* = 0.022). The diet effect was consistent considering MD adherence (IRR = 1.051, 95% CI 1.022–1.082, *p* < 0.001) (Table 2 and Supplementary Table 4). No significant interaction was between Age × Educational attainment (*p* = 0.53 and 0.40 for each level, respectively), Age × Physical Activity (*p* = 0.35) and Age × Healthy Diet (*p* = 0.22) (Supplementary Table 5).

### Central adiposity and VF

**FIGURE 3:**
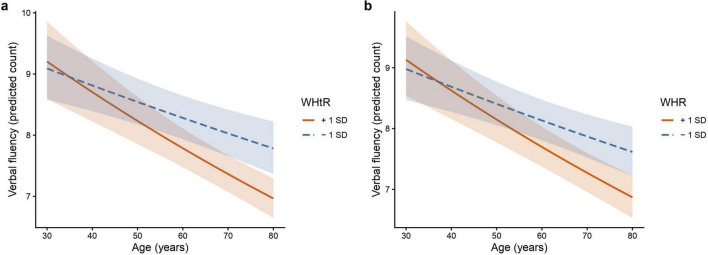
Interaction between age and Waist-Height ratio and Waist-Hip ratio on verbal fluency. Predicted verbal fluency scores according to age and WHtR **(a)** and WHR **(b)**. Results are adjusted for sex, physical activity, smoking status, healthy diet, education level, blood pressure, cholesterol values, and blood glucose. Shaded areas represent 95% confidence intervals. SD, standard deviation.

To examine the association between central adiposity and VF, we considered the fully adjusted model replacing BMI with WHtR and WHR. Higher central adiposity was associated with lower VF, both considering WHtR (IRR = 0.973, 0.960–0.986, *p* < 0.001) and WHR (IRR = 0.979, 95% CI 0.965–0.993, *p* = 0.003) (Table 3 and Supplementary Table 6). We next tested whether central adiposity moderated the association between age and verbal fluency, considering the Age × WHtR and Age × WHR interaction. The interaction term was lower than 1 and statistically significant both for WHtR (IRR = 0.985, 95% CI: 0.973–0.997, *p* = 0.014) and WHR (IRR 0.986, 95% CI 0.973–0.998, *p* = 0.021) indicating that the association between age and VF was moderated by central adiposity, with a steeper age-related reduction in individuals with higher levels of central adiposity (Figure 3). In Johnson-Neyman analysis the age effect was significant across the majority of participants both for WHtR and WHR values (Supplementary Figure 2). We observed a non-linear age × WHtR interaction (edf = 3.37, *p* = 0.001), whereas the age × WHR interaction was not significant (edf = 1.01, *p* = 0.16) (Supplementary Figure 3 and Supplementary Table 7).

**TABLE 3:** Association of central adiposity with verbal fluency and age interaction.

Variables	WHtR		WHtR interaction		WHR		WHR interaction	
	Estimate (95% CI)	*P*-value	Estimate (95% CI)	*P*-value	Estimate (95% CI)	*P*-value	Estimate (95% CI)	*P*-value
Age*	0.950 (0.937–0.964)	<0.001	0.949 (0.935–0.962)	<0.001	0.948 (0.934–0.961)	<0.001	0.947 (0.934–0.960)	<0.001
Education (9–16 yr)	1.229 (1.179–1.282)	<0.001	1.224 (1.174–1.276)	<0.001	1.234 (1.184–1.287)	<0.001	1.231 (1.181–1.283)	<0.001
Education (≥17 yr)	1.333 (1.277–1.392)	<0.001	1.329 (1.273–1.388)	<0.001	1.342 (1.286–1.401)	<0.001	1.339 (1.283–1.398)	<0.001
Sex (male)	0.991 (0.966–1.017)	0.483	0.989 (0.964–1.015)	0.396	1.002 (0.974–1.030)	0.901	1.003 (0.975–1.032)	0.821
Physically active (HM)	1.032 (1.006–1.059)	0.016	1.032 (1.005–1.059)	0.018	1.039 (1.012–1.066)	0.004	1.038 (1.011–1.065)	0.005
Non-smoker (HM)	1.013 (0.981–1.046)	0.431	1.013 (0.981–1.046)	0.430	1.011 (0.980–1.044)	0.481	1.012 (0.980–1.044)	0.471
Healthy diet (HM)	1.036 (1.010–1.062)	0.005	1.038 (1.012–1.064)	0.003	1.038 (1.013–1.064)	0.003	1.039 (1.013–1.065)	0.003
Blood pressure normal (HM)	1.016 (0.990–1.042)	0.242	1.016 (0.990–1.043)	0.227	1.022 (0.996–1.048)	0.100	1.022 (0.996–1.048)	0.097
Cholesterol normal (HM)	0.991 (0.966–1.015)	0.449	0.992 (0.967–1.016)	0.498	0.991 (0.967–1.016)	0.481	0.992 (0.968–1.017)	0.513
Blood glucose normal (HM)	1.053 (1.010–1.098)	0.016	1.048 (1.005–1.093)	0.029	1.056 (1.012–1.101)	0.012	1.050 (1.007–1.096)	0.023
Waist-height ratio*	0.973 (0.960–0.986)	<0.001	0.973 (0.960–0.986)	<0.001	–	–	–	–
Age × waist-height ratio*	–	–	0.985 (0.973–0.997)	0.014	–	–	–	–
Waist-hip ratio*	–	–	–	–	0.979 (0.965–0.993)	0.003	0.976 (0.962–0.990)	<0.001
Age × waist-hip ratio*	–	–	–	–	–	–	0.986 (0.973–0.998)	0.021

Incidence rate ratios for central adiposity and age × central adiposity interaction, from the fully adjusted negative binomial model. For health metrics, coefficients are referred to the positive value (in range). Est, Estimate; yr, years; HM, health metrics; AIC, Akaike Information Criterion; BIC, Bayesian Information Criterion. Estimates and confidence intervals are exponentiated.

*Age, WHtR and WHR were centered and scaled, coefficients are referred to a standard deviation increase. BMI and sleep quality not included in this model. CI, confidence interval.

### Sarcopenia and muscle function

Finally, we evaluated the associations between muscle parameters and verbal fluency performance. Among them, we observed a significant association between VF and CST performance: longer CST times were associated with a 3.6% lower VF (IRR = 0.964, 95% CI 0.951–0.977, *p* < 0.001), without any significant age interaction (*p* = 0.23) (Table 3 and Supplementary Table 8). Handgrip strength (*p* = 0.64), ASM (*p* = 0.77) and probable sarcopenia (*p* = 0.97) were not significantly associated with VF.

## Discussion

### Nonlinear association between age and VF

In this study, we evaluated the nonlinear association between age and VF performance, based on data from the Look Up 8+ study, on subject aged from 30 to 80 years. Moreover, we investigated the moderating role of central adiposity in the age-VF association. Our findings confirmed VF to be non-linearly associated with age, showing peak in mid-adulthood followed by a progressive reduction after the fifth decade of life. This underscores a methodological point, as most studies relying on linear regression model which may mischaracterize the timing of decline and miss the midlife plateau. Our findings align with recent evidence on the nonlinearity of brain aging: integrating MRI-derived and physiological data across four datasets (*n* = 19,300), Antal et al. (2025) found brain networks tend to destabilize throughout the lifespan, following a nonlinear trajectory, with evidence of specific temporal landmarks of brain aging starting during the 40’s (in the midlife), driven by dysregulated glucose homeostasis and partially reversible through metabolic (ketone-based) intervention.

The sharp change between 45 and 60 years of age is consistent with a recent study on VF performance, where authors found VF reaches peak around 40’s, with a further gradual decline in later adulthood (Konstantopoulos et al., 2026). Moreover, authors found that women tend to significantly perform better than men, especially after 40–45 years, while higher levels of educational attainment resulted associated with overall better performance in adults (Konstantopoulos et al., 2026). This aligns with findings from our cross-sectional analysis, where educational attainment exerted a strong and independent effect on VF and subjects with medium and high education showed 21.8% and 32.5% higher VF compared to subjects with low education.

### Educational attainment and cognitive reserve

A recent study investigated semantic and phonemic VF on a total of 144 adults aged 20–87, assessing influence of eight cognitive reserve factors, including educational attainment, on VF performance (Tremblay et al., 2025). Authors found that higher levels of education were associated with better VF performance and better global cognitive functioning: however, no clear protective effect of these factors against age−related decline emerged, and more longitudinal evidence is required on this topic.

The pattern whereby high education subjects reach their peak in VF performance results slightly earlier in our sample, with persistent superior performance throughout lifespan, consistent with the cognitive reserve hypothesis. Cognitive reserve is defined as the adaptability of cognitive processes that may help explaining differential susceptibility to brain function decline, including aging, pathology, or insult related mechanisms (Stern et al., 2020). According to this framework, education does not directly prevent cognitive aging but create a reserve of cognitive resources that allows brain to cope more effectively with age-related changes, maintaining better brain and cognitive functions for longer time. Coherently, it is established that higher education does not directly delay the onset of age-related cognitive decline but confers a persistently higher baseline performance (Clouston et al., 2020). However, cognitive reserve hypothesis has been subject to debate, as early education and socioeconomic status may shape development as passive markers of lifelong intellect rather than independent causal factors for late-life decline. Recent findings support a threshold model for dementia, with education establishing early cognitive differences that tend to persist into older age without altering the actual rate of dementia decline, challenging traditional cognitive reserve concepts (Lövdén et al., 2020). Given the cross-sectional design of this study, it is not possible to formally distinguish between a true cognitive-reserve mechanism and a threshold model in which education reflects early-life differences that persist, but do not alter the rate of decline.

### Cardiometabolic health metrics and verbal fluency

Among cardiometabolic HM, only healthy diet, PA and normal blood glucose values showed statistically significant independent associations with verbal fluency. Healthy diet was associated with a 4.4% higher verbal fluency score, coherently with previous evidence showing several foods typical of healthy nutritional regimens, including nuts, extra virgin olive oil (EVOO), dark chocolate, and eggs, enriched in specific nutrients such as mono-unsaturated fatty acids (MUFA), as associated with both improved memory and verbal fluency performance (Lopez de Coca et al., 2025). A recent study based on data from the 9th wave of the Survey of Health, Aging and Retirement in Europe (among older adults in 27 European countries and Israel), assessed the relationship between PA and indicators of cognitive function in older adults with osteoarthritis and moderate-severe pain: authors found moderate physical activity resulted associated with better VF performance in older adults (Ogrezeanu et al., 2025). Our study found PA associated with a 3.8% advantage in VF performance: being physically active represents a non-pharmacological strategy to support cognitive function in aging, acting through different molecular and neurofunctional pathways (Martín-Rodríguez et al., 2025). A key role is play by the muscle–brain axis, with skeletal muscle acting as an endocrine organ, exerting autocrine, paracrine, and endocrine effects (Hoffmann and Weigert, 2017). The endocrine function is linked to several molecular pathways between skeletal muscle and central nervous system, including myokines, exerting a mediating role in beneficial effects of exercise on the overall cognitive function (Vints et al., 2023). Subjects with normal values of blood glucose showed a 5% higher VF: this is consistent with existing evidence, with patients suffering from diabetes being at increased risk of cognitive decline and dementia (Biessels et al., 2006). Moreover, even among diabetic patients, those with uncontrolled disease tend to show a deteriorated cognitive function compared to subjects with optimal glycemic control, confirmed by a recent study based on an analysis on 79 patients suffering from type 2 diabetes mellitus (Alkethiri et al., 2021).

### Sex differences in verbal fluency

To note, our analysis showed no significant difference according to sex in VF, after accounting for age and education. Current literature regarding sex differences in cognitive functioning and in VF performance is controversial, as some evidence found male superiority in spatial tasks and a female advantage in verbal tasks, while other studies reported no significant sex differences in cognitive function (Andreano and Cahill, 2009; Lamé et al., 2025). In our sample, males and females showed similar VF values, suggesting that sex differences in verbal fluency may be attenuated when accounting for education.

### Central adiposity as a moderator of age-VF association

Findings from our moderation analysis showed that central adiposity moderated the association between age and VF with a steeper decrease in individuals with higher central adiposity. This is consistent with evidence on adiposity as one among several contributors in biological pathways through which aging impairs cognitive function: proposed mechanisms include visceral fat-induced chronic low-grade inflammation (with elevated IL-6, TNF-alpha, and C-reactive protein levels), adipokine dysregulation (increased leptin resistance), and insulin resistance with impaired cerebral glucose metabolism (Luchsinger et al., 2004; Shoelson et al., 2007). In addition, specific genes as the fat mass and obesity-associated (FTO) gene polymorphisms have been linked to a reduction of the frontal lobe volume and increased risk for Alzheimer disease in older subjects. Accordingly, obese and overweight subjects carrying FTO polymorphisms were found to perform worse on VF tests than non-carriers’ subjects (Benedict et al., 2011; Flores-Cordero et al., 2022).

Education and adiposity operate at different levels: the education-related gap largely reflects between-cohort differences in early-life cognitive investment rather than the biological rate of aging, whereas the steeper age-related decline at higher central adiposity points to a modifiable biological pathway superimposed on this background. Education sets the baseline level of performance, while central adiposity modulates how steeply performance differs across age.

### Muscle function versus muscle mass: the role of CST

Chair stand test performance emerged as the only significant covariate in the WHtR-VF pathway among muscle parameters: CST integrates lower-limb muscle power, neuromuscular coordination, and postural stability, which can be collectively impaired by overweight-related mechanical loading of the lower extremities. A plausible pathway from central adiposity to reduced functional lower-limb performance, potentially involving decreased cerebral perfusion and neuro-motor activation and coordination, is consistent with previous evidence linking gait speed and CST performance to dementia risk independently of muscle mass, with brain imaging data showing associations between lower-limb functional decline and gray matter reduction (Boyle et al., 2009; Buchman et al., 2007). ASM resulted as not associated with VF performance: this finding is consistent with the notion that muscle function (quality) rather than muscle quantity drives the link with cognitive performance across the whole lifespan. Abdominal adiposity may increase absolute muscle mass through weight-driven hypertrophy, yet this mass gain does not confer functional benefit, possibly reflecting concomitant intramuscular fat infiltration, reduced type II fiber proportion, and obesity-related mitochondrial dysfunction (Akazawa et al., 2017). Taken together, these findings suggest that functional rather than structural muscle indexes are associated with VF performance, with implications for potential intervention targeting. Indeed, formal mediation analysis on longitudinal data is warranted to test this pathway.

### Clinical and preventive implications

The Age × WHtR interaction on VF suggests that subjects with higher central adiposity present a steeper age-related decline in VF performance. This cross-sectional pattern suggests the 40–60 years span as a candidate for further longitudinal investigation on prevention strategies. Biologically, mechanisms underlying this moderation include adipose-driven and age-related neuroinflammation, accelerated cerebrovascular disease, and age-related immune-senescence affecting overall brain health and cognitive functioning (Miller et al., 2009; Shoelson et al., 2007; Uchida et al., 2024).

### Limitations

Several limitations of this study require acknowledgment. The cross-sectional design impose caution in the detection of trajectory differences that may only be observable longitudinally. Recruitment occurred in non-clinical community settings can be prone to selection bias, potentially skewing to population toward health-conscious and higher-education individuals: this may attenuate true population-level associations and limit the generalizability of these findings. Residual confounding by unmeasured variables cannot be ruled out. Due to the nature of the community-based screening design, several potentially relevant factors, such as cognitive reserve, socioeconomic status and occupation, depressive symptoms, neurological and cardiovascular comorbidities, medication use, sensory impairment, alcohol use, prior cognitive disorders, and lifetime PA history, were not recorded and therefore could not be included in the present models and could confound both the education and adiposity associations reported. Moreover, despite verbal fluency sensitivity in detecting cognitive impairment and dementia risk, it explores merely executive functions and may capture several aspects of the whole age-related brain function decline. In addition, the single-letter 30-s administration, while validated against the 60-s version (Kaufman et al., 2024), may be less reliable than the standard multi-letter 60-s protocol, subsequently underestimating individual variability. Longitudinal studies with multi-domain cognitive assessments, and repeated evaluations over time are required to confirm nonlinearity hypothesis of VF decline during lifespan, potential impact of education on this decline trajectory, and to confirm the observed moderation pathway and relative rates of cognitive decline over time, evaluating potential impact of preventive and actionable strategies.

## Conclusion

In conclusion, we found a non-linear association between age and VF, with peak performance in the fourth to fifth decade followed by progressively lower values at older ages. Educational attainment was a major determinant of performance, and no sex-specific differences emerged. Higher central adiposity was associated with a steeper age-related VF reduction, and we observed an association between lower-limb performance and VF, suggesting a potential functional pathway linking adiposity and cognitive aging.

## Data Availability

The datasets presented in this article are not readily available because of privacy concerns. Requests to access the datasets should be directed to the Look Up 8+ study group. Requests to access the datasets should be directed to francesco.landi@policlinicogemelli.it.
