# Supplementary material for: Nonlinear association between age and verbal fluency and moderation by adiposity: cross-sectional evidence from the Look Up 8+ study
Source: Front Aging Neurosci. 2026 Jul 29;18:1912213. doi: 10.3389/fnagi.2026.1912213 (PMC13461906; doi:10.3389/fnagi.2026.1912213)
Supplement: Supplementary file 1 [file Data_Sheet_1.pdf]

**Supplementary Materials**

**Summary.**


**Supplementary Table 1.** ........ 2
**Supplementary Table 2.** ........ 3
**Supplementary Figure 1.** ........ 4
**Supplementary Table 3.** ........ 5
**Supplementary Table 4.** ........ 5
**Supplementary Table 5.** ........ 6
**Supplementary Table 6.** ........ 7
**Supplementary Figure 2.** ........ 7
**Supplementary Table 7.** ........ 8
**Supplementary Figure 3.** ........ 8
**Supplementary Table 8.** ........ 9
**The Lookup 8+ Study Group** ........ 10
**STROBE checklist.** ........ 11

**Supplementary Table 1.**

**[A]**

| | **Gaussian** | | **Poisson** | | **Negative Binomial** | |
|---|---|---|---|---|---|---|
| | **Estimate (95% CI)** | **p-value** | **Estimate (95% CI)** | **p-value** | **Estimate (95% CI)** | **p-value** |
| **Age** | 0.925 (0.916 to 0.934) | <0.001 | 0.992 (0.991 to 0.993) | <0.001 | 0.992 (0.991 to 0.993) | <0.001 |

**[B]**

| | **Gaussian** | **Poisson** | **Negative Binomial** |
|---|---|---|---|
| **AIC** | 19750.5 | 19750.1 | 19556.6 |
| **BIC** | 19769.1 | 19762.5 | 19575.2 |
| **RMSE** | 3.71 | 3.72 | 3.72 |

**Supplementary Table 1.** Model coefficients (A) and model fit comparison (B) across regression families for the verbal fluency outcome. Mean VF was 10.1, and variance 14.7, indicating overdispersion.

A likelihood ratio test confirmed statistically significant overdispersion relative to a Poisson model ($\theta$=27.75, $p<0.001$), and the negative binomial model achieved substantially lower AIC than both Poisson and Gaussian alternatives. All outcome models were therefore estimated using a negative binomial GLM with log link. Estimate and confidence intervals are exponentiated. Age coefficient is referred to a 1yr change.

NB = negative binomial; AIC = Akaike Information Criterion; BIC = Bayesian Information Criterion: df = degrees of freedom.

**Supplementary Table 2.**
**[A]**

| | **Model 1 (GAM)** | **Model 2 (GAM)** | | **Model 3 (GAM)** | | **Model 3 (linear)** | |
|---|---|---|---|---|---|---|---|
| | **p-value** | **Estimate (95% CI)** | **p-value** | **Estimate (95% CI)** | **p-value** | **Estimate (95% CI)** | **p-value** |
| **s(Age)** | <0.001 | - | <0.001 | - | <0.001 | - | - |
| **Age (linear)** | - | - | - | - | - | 0.995 (0.994-0.996) | <0.001 |
| **Education (9-16yr)** | - | 1.232 (1.181-1.284) | <0.001 | 1.218 (1.168-1.271) | <0.001 | 1.233 (1.183-1.286) | <0.001 |
| **Education (≥17yr)** | - | 1.346 (1.289-1.405) | <0.001 | 1.326 (1.269-1.385) | <0.001 | 1.342 (1.286-1.401) | <0.001 |
| **Sex (Male)** | - | 0.978 (0.955-1.001) | 0.059 | 0.989 (0.963-1.015) | 0.387 | 0.987 (0.962-1.013) | 0.330 |
| **Physical Activity (HM: Active)** | - | - | - | 1.038 (1.012-1.065) | 0.005 | 1.039 (1.012-1.066) | 0.004 |
| **Smoke (HM: Non smoker)** | - | - | - | 1.013 (0.981-1.046) | 0.428 | 1.012 (0.981-1.045) | 0.452 |
| **Healthy Diet (HM: Healty Diet)** | - | - | - | 1.044 (1.018-1.070) | <0.001 | 1.039 (1.013-1.065) | 0.003 |
| **Blood Pressure (HM: Normal)** | - | - | - | 1.020 (0.994-1.047) | 0.133 | 1.023 (0.997-1.050) | 0.087 |
| **Cholesterol (HM: Normal)** | - | - | - | 0.992 (0.968-1.016) | 0.501 | 0.991 (0.967-1.016) | 0.481 |
| **Diabetes (HM: No diabetes)** | - | - | - | 1.050 (1.007-1.095) | 0.022 | 1.058 (1.014-1.103) | 0.009 |
| **BMI (HM: Normal)** | - | - | - | 1.023 (0.997-1.049) | 0.085 | 1.023 (0.997-1.049) | 0.085 |
| **Sleep Quality (HM: Good)** | - | - | - | 1.002 (0.977-1.028) | 0.891 | 1.002 (0.976-1.027) | 0.906 |

**[B]**

| | **Model 1 (GAM)** | **Model 2 (GAM)** | **Model 3 (GAM)** | **Model 3 (linear)** |
|---|---|---|---|---|
| **R2** | 0.070 | 0.113 | 0.119 | - |
| **AIC** | 19518.6 | 19335.2 | 19310.2 | 19331.3 |
| **BIC** | 19555.4 | 19389.1 | 19414.1 | 19418.0 |
| **RMSE** | 3.70 | 3.61 | 3.59 | 3.60 |

**Supplementary Table 2.** Model coefficients (A) and model fit comparison (B) of models evaluated. The fully adjusted model achieved lower AIC and BIC and were considered for further analysis. All outcome models were estimated using a negative binomial GLM with log link. Estimates and confidence intervals are exponentiated. For each HM measure, the reference is the “in range” value. Age coefficient is referred to a 1yr change. CI = Confidence Interval.

**Supplementary Figure 1.**

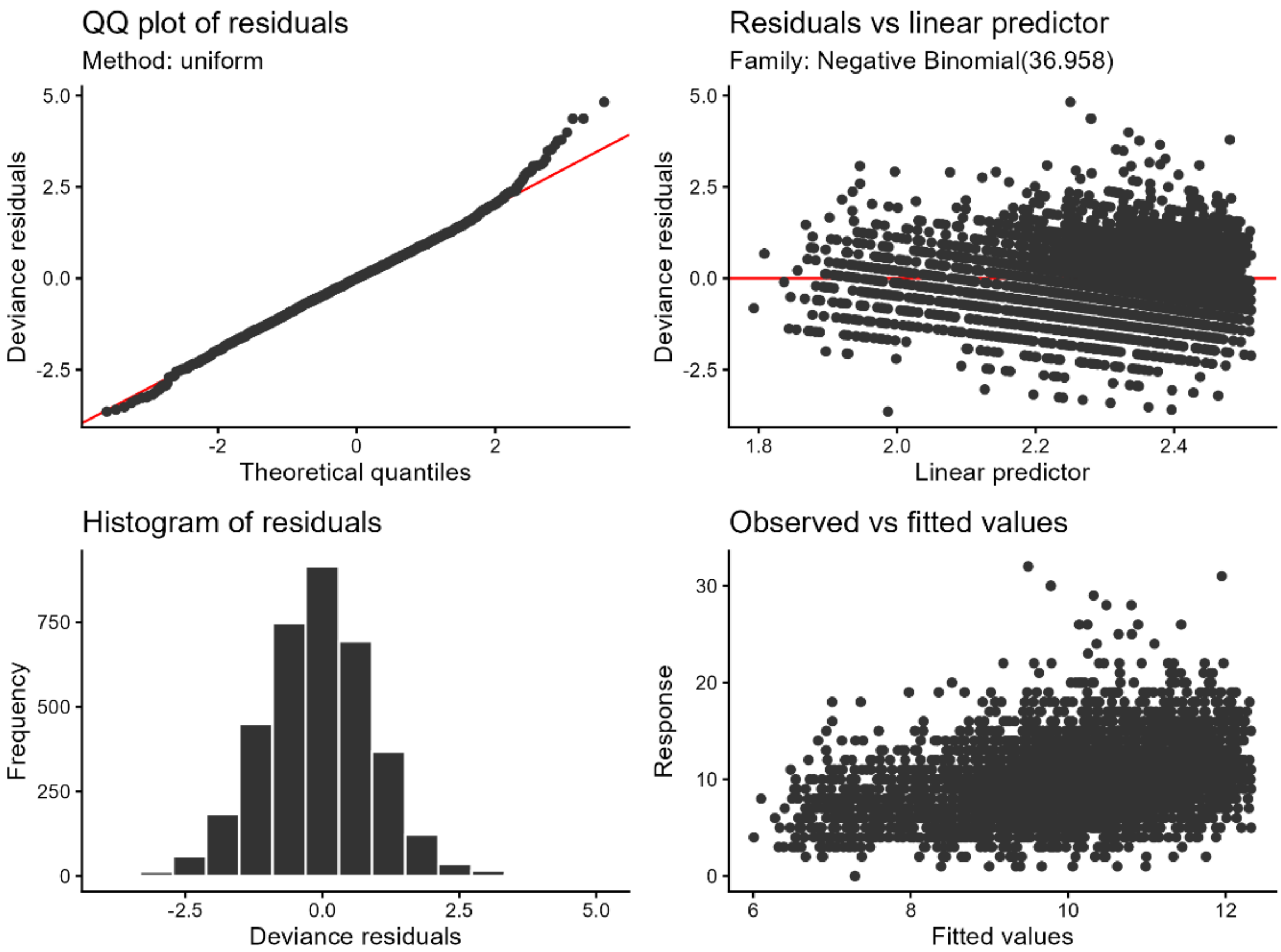


**Supplementary Figure 1.** Diagnostics for the fully adjusted model.

QQ-plot of residuals followed the reference line, with only a mild excess of large positive residuals in the upper tail. Residuals versus the linear predictor showed homogeneous scatter around zero with no heteroscedasticity. Residual histogram was symmetric. Observed versus fitted values were concordant, with the vertical spread reflecting the irreducible dispersion of the count outcome. Overall, no deviation from the negative binomial assumptions was evident.

Overdispersion, quantified as the Pearson dispersion statistic was 1.29 under Poisson distribution and negligible (1.02, θ = 37.0) under the negative binomial model.

**Supplementary Table 3.**
**[A]**

| | k | k' | edf | k-index | p-value |
|---|---|---|---|---|---|
| s(Age) | 5 | 4 | 3.01 | 0.91 | <0.001 |
| s(Age) | 10 | 9 | 3.36 | 0.91 | <0.001 |
| s(Age) | 15 | 14 | 3.39 | 0.91 | <0.001 |
| s(Age) | 20 | 19 | 3.40 | 0.91 | <0.001 |

**[B]**

| | s(Age) |
|---|---|
| worst | 0.313 |
| observed | 0.296 |
| estimate | 0.252 |

**[C]**

| Variable | GVIF | Df | GVIF^(1/(2*Df)) |
|---|---|---|---|
| Age | 1.417 | 1 | 1.19 |
| Education | 1.158 | 2 | 1.037 |
| Sex | 1.221 | 1 | 1.105 |
| Physical Activity | 1.114 | 1 | 1.055 |
| Smoke | 1.015 | 1 | 1.007 |
| Healthy Diet | 1.072 | 1 | 1.035 |
| Blood Pressure | 1.234 | 1 | 1.111 |
| Cholesterol | 1.119 | 1 | 1.058 |
| Diabetes | 1.043 | 1 | 1.021 |
| BMI | 1.142 | 1 | 1.069 |
| Sleep Quality | 1.021 | 1 | 1.011 |

**Supplementary Table 3. Additional model diagnostics. (A)** Basis-dimension sensitivity analysis for the age smooth. The fully adjusted model was refitted with increasing basis dimension. The edf stabilised at ≈3.4 and was unchanged across k, indicating that the default k=10 is appropriate. The k-index and its p-value did not improve with larger k. **(B)** Concurvity of the age smooth s(Age) with the remaining model terms, reported as worst, observed, and estimate. **(C)** Generalized variance inflation factors (GVIF) for the parametric covariates. All values were low, confirming the absence of multicollinearity. edf = effective degrees of freedom; k = specified basis dimension; k′ = effective basis dimension after identifiability constraints; GVIF = generalized variance inflation factor; Df = degrees of freedom.

**Supplementary Table 4.**

| | Healthy Diet (HM) | MEDI-LITE |
|---|---|---|
| **Number of subjects** | 3615 | 3499 |
| **AIC** | 19310.3 | 18704.4 |
| **BIC** | 19415.6 | 18809.2 |

**Supplementary Table 4.** Model fit comparison for diet. AIC = Akaike Information Criterion; BIC = Bayesian Information Criterion.

**Supplementary Table 5.**

| | Education interaction | | Physical activity interaction | | Healthy diet interaction | |
|---|---|---|---|---|---|---|
| | **Estimate (95% CI)** | **p-value** | **Estimate (95% CI)** | **p-value** | **Estimate (95% CI)** | **p-value** |
| **Age*** | 0.929 (0.890-0.970) | <0.001 | 0.937 (0.916-0.958) | <0.001 | 0.936 (0.916-0.955) | <0.001 |
| **Education (9-16yr)** | 1.219 (1.159-1.283) | <0.001 | 1.232 (1.182-1.285) | <0.001 | 1.233 (1.183-1.286) | <0.001 |
| **Education (≥17yr)** | 1.329 (1.264-1.399) | <0.001 | 1.341 (1.284-1.400) | <0.001 | 1.343 (1.286-1.402) | <0.001 |
| **Sex (Male)** | 0.987 (0.962-1.013) | 0.334 | 0.988 (0.963-1.014) | 0.352 | 0.987 (0.962-1.013) | 0.334 |
| **Physical Activity (HM: Active)** | 1.039 (1.012-1.066) | 0.004 | 1.038 (1.012-1.066) | 0.004 | 1.039 (1.012-1.066) | 0.004 |
| **Smoke (HM: Non smoker)** | 1.013 (0.981-1.045) | 0.436 | 1.012 (0.981-1.045) | 0.448 | 1.012 (0.980-1.045) | 0.467 |
| **Healthy Diet (HM: Healty Diet)** | 1.039 (1.014-1.065) | 0.002 | 1.039 (1.014-1.065) | 0.002 | 1.041 (1.015-1.067) | 0.002 |
| **Blood Pressure (HM: Normal)** | 1.023 (0.997-1.050) | 0.087 | 1.022 (0.996-1.049) | 0.093 | 1.023 (0.997-1.050) | 0.080 |
| **Cholesterol (HM: Normal)** | 0.991 (0.967-1.016) | 0.480 | 0.991 (0.967-1.016) | 0.480 | 0.991 (0.967-1.016) | 0.491 |
| **Diabetes (HM: No diabetes)** | 1.057 (1.014-1.102) | 0.010 | 1.057 (1.013-1.102) | 0.010 | 1.057 (1.014-1.103) | 0.010 |
| **BMI (HM: Normal)** | 1.023 (0.997-1.050) | 0.083 | 1.023 (0.997-1.049) | 0.086 | 1.023 (0.997-1.049) | 0.086 |
| **Sleep Quality (HM: Good)** | 1.002 (0.976-1.027) | 0.903 | 1.002 (0.977-1.028) | 0.889 | 1.002 (0.977-1.028) | 0.880 |
| **Age × Education (9-16yr)*** | 1.015 (0.969-1.063) | 0.525 | - | - | - | - |
| **Age × Education (≥17yr)*** | 1.020 (0.974-1.068) | 0.396 | - | - | - | - |
| **Age × Physical Activity*** | - | - | 1.012 (0.987-1.038) | 0.354 | - | - |
| **Age × Healthy Diet*** | - | - | - | - | 1.015 (0.991-1.041) | 0.221 |

**Supplementary Table 5.** Model coefficients for interaction terms. Estimates and confidence intervals are exponentiated. *Age was centred and scaled and coefficients are referred to a standard deviation increase.

**Supplementary Table 6.**

| | WHtR | WHtR interaction | WHR | WHR interaction |
|---|---|---|---|---|
| Number of subjects | 3615 | 3615 | 3615 | 3615 |
| AIC | 19316.8 | 19312.8 | 19323.2 | 19320.0 |
| BIC | 19397.3 | 19399.5 | 19403.7 | 19406.7 |
| RMSE | 3.60 | 3.59 | 3.60 | 3.60 |

**Supplementary Table 6.** Model fit comparison of models evaluated. AIC = Akaike Information Criterion; BIC = Bayesian Information Criterion.

**Supplementary Figure 2.**

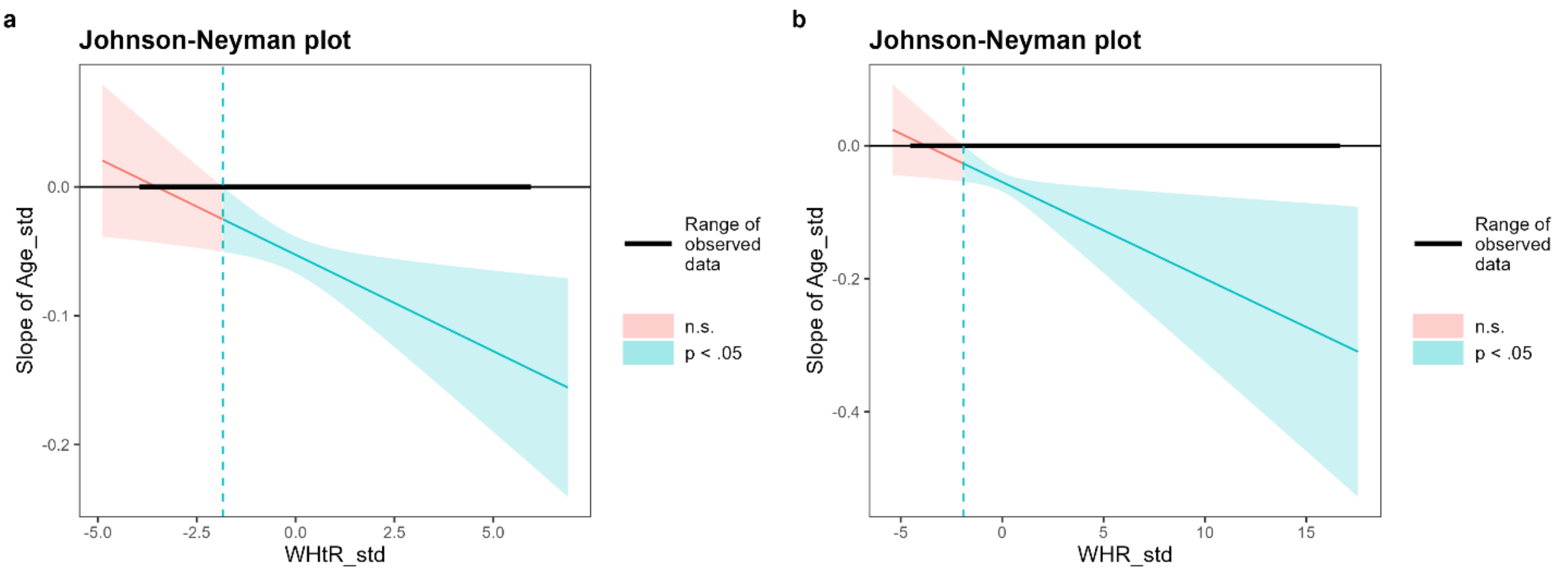


**Supplementary Figure 2.** Johnson-Neyman plot for the Age × WHtR (A) and Age × WHR (B) interaction on verbal fluency. The y-axis shows the simple slope of age (standardised) on verbal fluency across the range of WHtR and WHR(standardised). The teal region indicates values at which the age effect is statistically significant ($p<0.05$). The vertical dashed line marks the Johnson-Neyman threshold and the horizontal black bar indicates the range of observed values in the sample.

**Supplementary Table 7.**
**[A]**

| | WHtR (GAM interaction) | | WHR (GAM interaction) | |
|---|---|---|---|---|
| | edf | p-value | edf | p-value |
| s(Age)* | 2.94 | <0.001 | 3.27 | <0.001 |
| s(Age) × Waist-Height Ratio* | 3.37 | 0.001 | - | - |
| s(Age) × Waist-Hip Ratio* | - | - | 1.01 | 0.160 |

**[B]**

| | WHtR (GAM) | WHtR (GAM interaction) | WHR (GAM) | WHR (GAM interaction) |
|---|---|---|---|---|
| Number of subjects | 3615 | 3615 | 3615 | 3615 |
| AIC | 19297.3 | 19298.2 | 19302.8 | 19303.2 |
| BIC | 19395.0 | 19412.6 | 19400.3 | 19405.8 |
| RMSE | 3.58 | 3.58 | 3.59 | 3.59 |

**Supplementary Table 7.** Model results (A) and model fit comparison (B) for GAM models, without and with the non-linear age x central adiposity interaction. edf: Estimate; AIC = Akaike Information Criterion; BIC = Bayesian Information Criterion. *Age, WHtR and WHR were centred and scaled. CI = Confidence Interval.

**Supplementary Figure 3.**

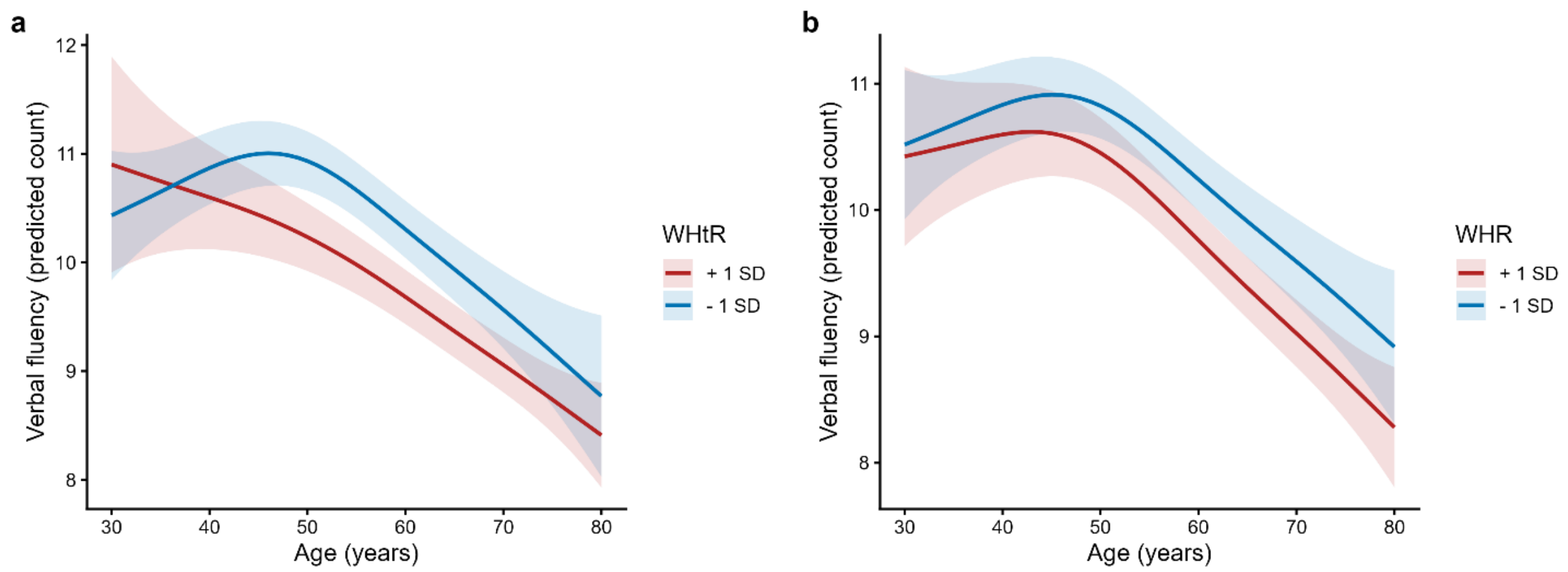


**Supplementary Figure 3.** Predicted verbal fluency scores according to age and WHtR (A) and WHR (B) for the GAM model including non-linear interaction. Predictions are averaged over the covariate distribution (sex, physical activity, smoking, healthy diet, education, blood pressure, cholesterol, blood glucose). Shaded areas represent 95% confidence intervals. SD = standard deviation.

**Supplementary Table 8.**

| | Handgrip | | Chair-stand test | | ASM | | Sarcopenia (EWGSOP2) | |
|---|---|---|---|---|---|---|---|---|
| | **Estimate (95% CI)** | **p-value** | **Estimate (95% CI)** | **p-value** | **Estimate (95% CI)** | **p-value** | **Estimate (95% CI)** | **p-value** |
| **Age*** | 0.951 (0.937-0.965) | <0.001 | 0.959 (0.945-0.973) | <0.001 | 0.951 (0.937-0.965) | <0.001 | 0.951 (0.938-0.965) | <0.001 |
| **Waist-Height Ratio*** | 0.973 (0.960-0.986) | <0.001 | 0.979 (0.966-0.993) | 0.003 | 0.972 (0.957-0.987) | <0.001 | 0.973 (0.960-0.986) | <0.001 |
| **Education (9-16yr)** | 1.229 (1.179-1.281) | <0.001 | 1.223 (1.174-1.276) | <0.001 | 1.229 (1.179-1.282) | <0.001 | 1.229 (1.179-1.281) | <0.001 |
| **Education (≥17yr)** | 1.333 (1.277-1.392) | <0.001 | 1.325 (1.270-1.384) | <0.001 | 1.333 (1.277-1.392) | <0.001 | 1.333 (1.277-1.392) | <0.001 |
| **Sex (Male)** | 0.985 (0.950-1.021) | 0.409 | 0.985 (0.960-1.011) | 0.250 | 0.984 (0.937-1.035) | 0.538 | 0.992 (0.967-1.017) | 0.523 |
| **Physical Activity (HM: Active)** | 1.032 (1.006-1.059) | 0.017 | 1.025 (0.999-1.052) | 0.059 | 1.032 (1.006-1.059) | 0.017 | 1.032 (1.005-1.059) | 0.018 |
| **Smoke (HM: Non smoker)** | 1.013 (0.981-1.046) | 0.428 | 1.013 (0.981-1.045) | 0.433 | 1.013 (0.981-1.045) | 0.433 | 1.013 (0.981-1.046) | 0.428 |
| **Healthy Diet (HM: Healty Diet)** | 1.036 (1.011-1.062) | 0.005 | 1.037 (1.011-1.062) | 0.004 | 1.036 (1.010-1.062) | 0.005 | 1.036 (1.011-1.062) | 0.005 |
| **Blood Pressure (HM: Normal)** | 1.016 (0.990-1.043) | 0.234 | 1.015 (0.989-1.042) | 0.250 | 1.016 (0.990-1.043) | 0.241 | 1.016 (0.990-1.043) | 0.235 |
| **Cholesterol (HM: Normal)** | 0.991 (0.967-1.015) | 0.452 | 0.991 (0.967-1.015) | 0.462 | 0.990 (0.966-1.015) | 0.443 | 0.991 (0.967-1.015) | 0.457 |
| **Diabetes (HM: No diabetes)** | 1.052 (1.009-1.098) | 0.017 | 1.047 (1.004-1.092) | 0.032 | 1.053 (1.010-1.098) | 0.017 | 1.052 (1.009-1.097) | 0.018 |
| **Handgrip strength*** | 1.004 (0.987-1.022) | 0.642 | - | - | - | - | - | - |
| **Chair stand test performance*** | - | - | 0.964 (0.951-0.977) | <0.001 | - | - | - | - |
| **Appendicular Skeletal Muscle Mass*** | - | - | - | - | 1.004 (0.978-1.031) | 0.765 | - | - |
| **Probable Sarcopenia** | - | - | - | - | - | - | 0.971 (0.924-1.021) | 0.249 |

**Supplementary Table 8.** Model coefficients of models evaluated. Estimates and confidence intervals are exponentiated. *Age, WHtR, handgrip strength, chair stand test performance and ASM were centred and scaled. BMI and sleep quality not included in this model. CI = Confidence Interval.

**The Lookup 8+ Study Group**

The Lookup 8+ Study Group is composed as follows: Francesco Landi [a,b], Roberto Bernabei [a], Emanuele Marzetti [a,b], Riccardo Calvani [a,b], Luca Mariotti [a,b], Stefano Cacciatore [a,b], Hélio José Coelho-Junior [a,b], Francesca Ciciarello [b], Vincenzo Galluzzo [b], Anna Maria Martone [a,b], Anna Picca [b,c], Andrea Russo [b], Sara Salini [b], Matteo Tosato [b], Gabriele Abbatecola [a], Clara Agostino [a], Fiorella Ambrosio [a], Francesca Banella [a], Carolina Benvenuto [a], Damiano Biscotti [b], Vincenzo Brandi [b], Maria Modestina Bulla [a], Caterina Casciani [a], Lucio Catalano [b], Camilla Cocchi [a,b], Giuseppe Colloca [b], Federica Cucinotta [a], Emanuela D'Angelo [b], Mariaelena D'Elia [b], Federica D'Ignazio [a,b], Daniele Elmi [a], Marta Finelli [a], Francesco Pio Fontanella [a], Domenico Fusco [b], Ilaria Gattari [a], Giordana Gava [a,b], Tommaso Giani [a,b], Giulia Giordano [a,b], Rossella Giordano [a,b], Francesca Giovanale [a], Simone Goracci [a], Silvia Ialungo [a,b], Rosangela Labriola [a], Elena Levati [a,b], Myriam Macaluso [a,b], Luca Marrella [a], Claudia Massaro [a,b], Rossella Montenero [a,b], Maria Vittoria Notari [a], Maria Paudice [a], Martina Persia [a], Flavia Pirone [a], Simona Pompei [b], Rosa Ragozzino [a], Carla Recupero [a], Antonella Risoli [a], Stefano Rizzo [a], Daria Romaniello [a], Giulia Rubini [a], Barbara Russo [a], Stefania Satriano [a], Giulia Savera [a], Elisabetta Serafini [b], Annalise Serra Melechì [a], Francesca Simeoni [a], Sofia Simoni [a,b], Chiara Taccone [a], Elena Tagliacozzi [a], Roberta Terranova [a], Salvatore Tupputi [a], Matteo Vaccarella [a], Emiliano Venditti [a], Chiara Zanchi [a], Maria Zuppardo [a]

[a] Department of Geriatrics, Orthopedics and Rheumatology, Università Cattolica del Sacro Cuore, L.go F. Vito 1, 00168 Rome, Italy.

[b] Fondazione Policlinico Universitario "Agostino Gemelli" IRCCS, L.go A. Gemelli 8, 00168 Rome, Italy.

[c] Department of Medicine and Surgery, LUM University, Strada Statale 100 km 18, 70100 Casamassima, Italy.

**STROBE checklist.**

| | Item No | Recommendation | Page No |
|---|---|---|---|
| **Title and abstract** | 1 | (*a*) Indicate the study's design with a commonly used term in the title or the abstract | 1 |
| | | (*b*) Provide in the abstract an informative and balanced summary of what was done and what was found | 2 |
| **Introduction** | | | |
| Background/rationale | 2 | Explain the scientific background and rationale for the investigation being reported | 3 |
| Objectives | 3 | State specific objectives, including any prespecified hypotheses | 3 |
| **Methods** | | | |
| Study design | 4 | Present key elements of study design early in the paper | 4 |
| Setting | 5 | Describe the setting, locations, and relevant dates, including periods of recruitment, exposure, follow-up, and data collection | 4 |
| Participants | 6 | (*a*) Give the eligibility criteria, and the sources and methods of selection of participants | 4 |
| Variables | 7 | Clearly define all outcomes, exposures, predictors, potential confounders, and effect modifiers. Give diagnostic criteria, if applicable | 4 |
| Data sources/ measurement | 8* | For each variable of interest, give sources of data and details of methods of assessment (measurement). Describe comparability of assessment methods if there is more than one group | 4 |
| Bias | 9 | Describe any efforts to address potential sources of bias | 4-5 |
| Study size | 10 | Explain how the study size was arrived at | NA |
| Quantitative variables | 11 | Explain how quantitative variables were handled in the analyses. If applicable, describe which groupings were chosen and why | 4-5 |
| Statistical methods | 12 | (*a*) Describe all statistical methods, including those used to control for confounding | 4-5 |
| | | (*b*) Describe any methods used to examine subgroups and interactions | 5 |
| | | (*c*) Explain how missing data were addressed | 5 |
| | | (*d*) If applicable, describe analytical methods taking account of sampling strategy | 5 |
| | | (*e*) Describe any sensitivity analyses | NA |
| **Results** | | | |
| Participants | 13* | (a) Report numbers of individuals at each stage of study—eg numbers potentially eligible, examined for eligibility, confirmed eligible, included in the study, completing follow-up, and analysed | 6 |
| | | (b) Give reasons for non-participation at each stage | NA |
| | | (c) Consider use of a flow diagram | NA |
| Descriptive data | 14* | (a) Give characteristics of study participants (eg demographic, clinical, social) and information on exposures and potential confounders | 6 |
| | | (b) Indicate number of participants with missing data for each variable of interest | NA |
| Outcome data | 15* | Report numbers of outcome events or summary measures | 6-7 |
| Main results | 16 | (*a*) Give unadjusted estimates and, if applicable, confounder-adjusted estimates and their precision (eg, 95% confidence interval). Make clear which confounders were adjusted for and why they were included | 6-7 |
| | | (*b*) Report category boundaries when continuous variables were categorized | 6-7 |
| | | (*c*) If relevant, consider translating estimates of relative risk into absolute risk for a meaningful time period | NA |
| Other analyses | 17 | Report other analyses done—eg analyses of subgroups and interactions, and sensitivity analyses | 6-7 |
| **Discussion** | | | |
| Key results | 18 | Summarise key results with reference to study objectives | 8-10 |
| Limitations | 19 | Discuss limitations of the study, taking into account sources of potential bias or imprecision. Discuss both direction and magnitude of any potential bias | 10 |
| Interpretation | 20 | Give a cautious overall interpretation of results considering objectives, limitations, multiplicity of analyses, results from similar studies, and other relevant evidence | 8-10 |
| Generalisability | 21 | Discuss the generalisability (external validity) of the study results | 10 |
| **Other information** | | | |
| Funding | 22 | Give the source of funding and the role of the funders for the present study and, if applicable, for the original study on which the present article is based | 16 |
